# Understanding and practices of over-the-counter drugs for self-care among students of different medical faculties in a low-and middle-income country

**DOI:** 10.1097/MS9.0000000000003208

**Published:** 2025-03-19

**Authors:** Sitaram Khadka, Rakshya KC, Asmita Luitel, Suchit Thapa Chhetri, Sanjeev Gurung, Prem Prasad Panta, Seema Waiba

**Affiliations:** a Shree Birendra Hospital; Nepalese Army Institute of Health Sciences, Kathmandu, Nepal; b Valley College of Technical Sciences, Kathmandu, Nepal; c Nepalese Army Institute of Health Sciences, Kathmandu, Nepal; d KIST Medical College, Lalitpur, Nepal

**Keywords:** health sciences, health science students, low-and middle-income countries, over-the-counter drugs, self-medication

## Abstract

**Introduction:**

Over-the-counter (OTC) drugs play a critical role in self-care, markedly in low- and middle-income countries (LMICs) like Nepal. Understanding and practices related to OTC drug use among health science students are crucial, given their role in the future of healthcare. This study was conducted with the objective to compare and assess the understanding and practices of OTC drugs for self-care among medical, pharmacy, and nursing students and evaluate the impact of their education on these practices.

**Methods:**

A multicenter comparative cross-sectional study was conducted across three health science colleges affiliated with different universities in Nepal. A total of 129 health science students from medicine (77), pharmacy (23), and nursing (29) faculties were selected. Understanding and practice scores were analyzed, and socio-demographic variables were examined for associations with OTC drug use.

**Results:**

Most students exhibited moderate to poor understanding and practices regarding OTC drug use. Understanding scores were comparable across faculties, with medical students showing the highest proportion of good understanding (7.8%). Practice scores were highest among pharmacy students (4.34%). A significant number of students self-medicated without consulting healthcare professionals and often used OTC drugs due to convenience. There were no statistically significant differences in understanding and practice of OTC based on socio-demographic factors, including faculties.

**Conclusion:**

The study highlighted a general lack of understanding and inadequate practices related to OTC drugs among health science students. These findings underscore the need for improved education and regulation to promote responsible self-medication practices.

**HIGHLIGHTS**

- Self-medication is prevalent among medical students in LMICs despite the differences in faculties.
- There is a general lack of understanding and inadequate practices related to OTC drugs among medical students.
- As future healthcare professionals and role models for their peers, medical students have a significant responsibility to set an example in the responsible use of medicines.
- Improved education, stringent legislation, and the effective implementation of programs and guidelines to mitigate irresponsible self-medication practices are highly needed.

## Introduction

The World Health Organization (WHO) defines over-the-counter (OTC) drugs as medicinal products that can be sold without a prescription^[1]^. These drugs provide both preventive care and treatment for minor ailments, including headaches, heartburn, colds, musculoskeletal pain, and more^[2]^. Self-medication involves using medicines to treat self-diagnosed conditions or symptoms, as well as the intermittent or regular use of prescribed medicines for chronic or recurring conditions^[3]^. This practice may include obtaining medications without a valid prescription, using old prescriptions, sharing medicines among family and friends, or consuming leftover medications stored at home^[4]^. It entails the use of medication without professional guidance regarding its indication, dosage, and duration^[4]^. Self-medication is prevalent in low- and middle-income countries (LMICs), driven by factors, such as patient dissatisfaction with government healthcare facilities and physicians, the high cost of prescription drugs, limited education, unregulated medicine distribution, untrained pharmacy personnel, and inadequate doctor–patient ratios in healthcare systems^[5,6]^.

Responsible self-medication involves using OTC drugs with adequate knowledge about their indications, dosages, potential side effects, interactions, precautions, warnings, duration of use, and recognizing when to seek medical advice^[7]^. Globally, healthcare systems have integrated responsible self-medication practices along with educational and awareness initiatives, taking into account socioeconomic factors and healthcare resources^[8]^. Self-care is a naturalistic approach to decision-making that focuses on the prevention and management of chronic illnesses and encompasses three core elements: self-care management, self-care monitoring, and self-care maintenance^[9]^. Self-medication is considered a key component of self-care^[10]^. It is increasingly recognized that appropriate self-medication can benefit patients, healthcare providers, the pharmaceutical industry, and governments alike^[11]^. In recent years, individuals have shown a growing desire to take greater responsibility for their health by seeking reliable information from reputable sources to help make informed decisions about their healthcare^[12]^.

In LMICs, the most commonly self-medicated drugs include non-steroidal anti-inflammatory drugs (NSAIDs), antacids, acid inhibitors, oral rehydration solutions, and antibiotics^[13]^. However, the misuse or abuse of OTC drugs, in the general population and among health science students, raises ethical and clinical concerns^[14,15]^. The widespread promotion of drugs and extensive media exposure contribute to increased risks, leading to challenges such as inaccurate self-diagnosis, harmful drug interactions, and the use of medications for unintended purposes. Improper self-medication poses various risks, including adverse drug reactions (ADRs), incorrect drug selection, delayed diagnosis, drug dependency, drug–drug and drug–food interactions, and the risk of poisoning or overdose^[16]^. Despite these risks, the market for OTC drugs continues to expand, and their use remains widespread^[17]^.

When used appropriately, self-medication can help sustain limited medical resources for more serious conditions, reduce the load on healthcare facilities, and save both time and money for patients^[18]^. In recent years, the self-medication of OTC drugs has become increasingly common due to their easy accessibility. This practice is particularly relevant among health science students, who will eventually become healthcare providers and must counsel patients on the aids and perils of self-medication. Health science students are expected to have more prevalence of self-medication compared to the general population due to their greater exposure to medical knowledge^[18]^. Their health-related knowledge, attitudes, and behaviors not only influence their health but also impact the healthcare system as a whole^[8]^.

The global prevalence of self-medication is high, ranging from 32.5% to 81.5%^[19]^. In European countries, the prevalence is around 68%, while in LMICs like Nepal and India, it stands at 59% and 31%, respectively^[19]^. However, there is limited research on the understanding and practices related to OTC drugs for self-care among health science students. This lack of information highlights the need to investigate how health science students perceive and utilize OTC drugs, which are readily accessible. Increased self-medication among health science students could lead to delayed treatment of serious conditions and the masking of critical symptoms through the use of non-prescription drugs.

Therefore, this study aims to compare and assess the extent of health science students’ understanding and practice of OTC drugs for self-care and to determine how their education and training influence these practices.

## Methods

### Ethical approval

The research was carried out according to the Declaration of Helsinki. Ethical clearance for this study was acquired from the Institutional Review Committee of the Nepalese Army Institute of Health Sciences (IRC-NAIHS) with registered number 1073. The ethical clearance was also obtained from the Nobel College with reference number 080/081.292. Informed consent to participate in the study was also taken from participants.

### Study design

A multicenter comparative cross-sectional study design was applied concerning the STROCSS (strengthening the reporting of cohort, cross-sectional, and case-control studies in surgery) 2021 criteria (Supplementary file 1, http://links.lww.com/MS9/A770)^[20]^. It was registered in the ResearchRegistry 10617.

### Study setting

The study was conducted across several colleges affiliated with three different universities that offer health science education in the Kathmandu Valley, Nepal from 14 June 2024 to 19 August 2024. These included the Nepalese Army Institute of Health Sciences (NAIHS) affiliated with Tribhuvan University for medical students, the Valley College of Technical Sciences (VCTS) affiliated with Purbanchal University for pharmacy students, and the Nobel College affiliated with Pokhara University for nursing students.

Nepal is classified as an LMIC. Kathmandu, the capital city of Nepal, comprises Kathmandu, Lalitpur, and Bhaktapur districts. The city is the central hub for academic, health, and administrative services, attracting students from across the country who gain admission through the common entrance examination conducted by the Medical Education Commission (MEC). Thus, the selected study area represents the health science institutes of Nepal.

### Study population and sample size

The study population consisted of final-year health science students from different faculties, including medicine (MBBS) students from NAIHS affiliated with Tribhuvan University, pharmacy (BPharm) students from VCTS affiliated with Purbanchal University, and nursing (BSc Nursing) students from Nobel College affiliated with Pokhara University in Kathmandu.

The sample size was calculated using Cochrane’s formula^[21]^:

***n* = *Z*^2^ × *p* × *q*/*d^2^*** = 384.16−385,

where *n* = required sample size; *Z* = 1.96 at 95% confidence level; *p* = estimated prevalence rate (50%); *q* = probability of non-occurrence of *p* (100% – *p*); and *d* = allowable error (5%).

A 5% allowable error was chosen to ensure a balance between precision and practical constraints. This prevalence rate was selected because it represents the maximum variability, providing the largest possible sample size.

Given that the total number of final-year health science students in the selected colleges (*N*) was 168, the adjusted sample size (*n’*) was calculated using the formula:

***n’* = *n*/[1 + (*n* – 1)/*N*] =** 117.

Considering a 10% non-response rate (11.7), the sample size (*n’*) was 128.89−129 (*n’* = 129).

For data collection using stratified random sampling, the proportionate stratified random sampling formula was applied:

***n*_h_ = (*N*_h_/*N*) × n,**

where *n*_h_ = sample size for *h*^th^ stratum; *N*_h_ = population size for h^th^ stratum; *N* = size of the entire population; *n* = size of the entire sample.

- For 100 medicine students, the required sample size (*n*_m_) = (129/ 168) **×** 100 = 76.78 ≈ 77.
- For 30 pharmacy students, the required sample size (*n*_p_) = (129/ 168) **×** 30 = 23.03 ≈ 23.
- For 38 nursing students, the required sample size (*n*_n_) = (129/ 168) **×** 38 = 29.17 ≈ 29.

Thus, a total of 77 medical, 23 pharmacy, and 29 nursing students (total sample size = 129) were selected for the study.

### Sampling technique

All health science universities and colleges were identified from the Ministry of Education and Sports, as well as from the Medical, Pharmacy, and Nursing Councils of Nepal, and purposively three programs were selected.

The sampling frame of the final year students of each program was prepared and representative samples were taken from the different strata using sampling fraction. Every third participant was selected from the different programs using systematic random sampling.

### Study tool and data collection

A structured questionnaire was prepared through an extensive literature review for the data collection. It comprised three sections, including socio-demographic data, understanding, and practice toward self-care using OTC drugs.

Pre-testing was done for the reliability of the tools among the 10% of the total sample size which was not included in the final data set. The reliability analysis yielded a Cronbach alpha value of 0.73. Therefore, the tools were used for the data collection.

A self-administered survey method was then employed for the data collection process.

### Data management and statistical analysis

Data were entered into MS Excel and subsequently exported to SPSS version 23 for analysis.

The understanding and practice items consist of ten questions (maximum score of 50) each. The statements in line with the concept of understanding and/or practices were graded 5 points for strongly agree and 1 point for strongly disagree, and accordingly rest responses of disagree, neutral, and agree were graded 2, 3, and 4 points, respectively. The reverse coding was done for the statements opposite to the view of understanding and/or practices.

Scores for understanding and practice were categorized into “good” (≥80% of 50 = ≥40) and “moderate-to-poor” (<80% of 50 = <40) based on modified Bloom’s cut-off criteria^[22]^.

Socio-demographic data were displayed as frequencies and proportions. Binary logistic regression was executed to determine the predictors of understanding and practice of OTC drugs. A box plot was used to illustrate the understanding and practice scores distribution across different factors.

Spearman’s rank correlation coefficient was applied to assess relationships between understanding and practice scores. *P*-value < 0.05 was considered statistically significant.

## Results

The study encompassed 129 students: 77 (59.75%) were from medicine, 23 (17.8%) were from pharmacy, and 29 (22.5%) were from nursing program. Additionally, 53.5% of the respondents were male and most were medicine (68.8%) and pharmacy (65.2%) students in their mid-20s (Table 1).

**Table 1:** Socio-demographic characteristics of the participants (*N* = 129)

S.N.	Characteristics	Medicine (*N*_1_ = 77) No. (%)	Pharmacy (*N*_2_ = 23) No. (%)	Nursing (*N*_3_ = 29) No. (%)
1	Age (years)			
	Early 20s: 20–23	15 (19.5)	3 (13)	14 (48.3)
	Mid 20s: 24–26	53 (68.8)	15 (65.2)	14 (48.3)
	Late 20s: 27–29	9 (11.7)	5 (21.7)	1 (3.4)
	30 and above	-	-	-
2	Gender			
	Male	52 (67.5)	17 (73.9)	-
	Female	25 (32.5)	6 (26.1)	29 (100)
3	Living status			
	With family	48 (62.3)	15 (65.2)	21 (72.4)
	Alone	29 (37.7)	8 (34.8)	8 (27.6)
4	University			
	Tribhuvan University	77 (100)	-	-
	Purbanchal University	-	23(100)	-
	Pokhara University	-	-	29 (100)
5	Faculty			
	Medicine	77 (100)	-	-
	Pharmacy	-	23 (100)	-
	Nursing	-	-	29 (100)
6	Religion			
	Hindu	70 (90.9)	22 (95.7)	27 (93.1)
	Buddhist	2 (2.6)	-	1 (3.4)
	Christian	1 (1.3)	-	1 (3.4)
	Muslim	2 (2.6)	1 (4.3)	-
	Others	2 (2.6)	-	-
7	Monthly household income			
	<30 000	10 (13)	5 (21.7)	6 (20.7)
	30 000–50 000	17 (22.1)	9 (39.1)	6 (20.7)
	51 000–100 000	35 (45.5)	7 (30.4)	14 (48.3)
	>100 000	15 (19.5)	2 (8.7)	3 (10.3)
8	One of the parent’s profession			
	Medical	10 (13)	6 (26.1)	5 (17.2)
	Non-medical	67 (87)	17 (73.9)	2 (82.8)
9	Permanent address			
	Koshi Pradesh	6 (7.8)	2 (8.7)	3 (10.3)
	Madhesh Pradesh	20 (26)	9 (39.1)	1 (3.4)
	Bagmati Pradesh	26 (33.8)	6 (26.1)	15 (51.7)
	Gandaki Pradesh	11 (14.3)	1 (4.3)	1 (3.4)
	Lumbini Pradesh	6 (7.8)	5 (21.7)	3 (10.3)
	Karnali Pradesh	4 (5.2)	-	4 (13.8)
	Sudurpaschim Pradesh	2 (2.6)	-	2 (6.9)
	India	2 (2.6)	-	-
10	Area (permanent address)			
	Urban	42 (54.5)	14 (60.9)	22 (75.9)
	Rural	35 (45.5)	9 (39.1)	7 (24.1)

The majority of participants exhibited moderate to poor understanding of OTC medications across all faculties. Only a small percentage of students from medicine (7.8%), pharmacy (4.3%), and nursing (3.4%) demonstrated good understanding. Practice scores also reflected moderate to poor practices across all faculties. Good practice was observed in a minority of students, with pharmacy having the highest proportion (4.34%) (Table 2).

**Table 2:** Understanding and practice scores of the participants

S.N.	Particulars	Characteristics	Scores/Values		
			Medicine	Pharmacy	Nursing
1.	Understanding	Median(min-max)	35 (25–45)	36 (26–41)	33 (27–42)
		Mean ± SD	34.77 ± 3.7	34.57 ± 3.6	3345 ± 3.02
		Q1-Q3	33-37	31-36	31-35
		Good understanding	7.8% (6/77)	4.3% (1/23)	3.4% (1/29)
		Moderate to poor understanding	92.2% (71/77)	95.6% (22/23)	96.6% (28/29)
2.	Practice	Median (min–max)	27 (10–46)	24 (15–40)	28 (18–44)
		Mean ± SD	27.31 ± 5.9	24.4 ± 6.1	27.9 ± 5.5
		Q1–Q3	24–30	20–27	24–30
		Good practice	1.3% (1/77)	4.34% (1/23)	3.4% (1/29)
		Moderate-to-poor practice	98.7% (76/77)	95.66% (22/23)	96.6% (28/29)

In the understanding-related questions, the majority of participants across all faculties strongly agreed on the importance of stopping OTC drug use immediately if side effects are observed (medicine: 55.8%, pharmacy: 47.8%, nursing: 58.6%). A high proportion of pharmacy (52.2%) and medicine (57.1%) students agreed OTC drugs as cost-effective, while nursing students mostly remained neutral (62.1%). Additionally, a large proportion of medicine (63.6%) and pharmacy (43.5%) strongly agreed that irrational OTC use can be harmful (Supplementary Table A, http://links.lww.com/MS9/A770).

In the practice-related questions, most students agreed to self-medicate with OTC drugs (medicine: 85.7%, pharmacy: 91.3%, nursing: 82.8%). Over half of the medicine (70.1%) and pharmacy students (69.6%) used OTCs for minor ailments compared to nursing students (48.3%). Many did not consult a practitioner before self-medicating (medicine: 54.5%, pharmacy: 52.2%, and nursing: 37.9%). Regular checking of expiry dates was very low (medicine: 5.2%, pharmacy: 8.7%, and nursing: 20.7%) (Supplementary Table B, http://links.lww.com/MS9/A770).

Participants up to 23 years old showed 87.5% moderate to poor understanding, while those above 23 showed 95.9%. Odds of good understanding were 3.32 times higher in the younger group (odds ratio [OR]: 0.301, 95% confidence interval [CI]: 0.071–1.182, *P =* 0.088). Females had a slightly better understanding (6.7%) than males (5.8%), with 1.16 times higher odds of good understanding (OR: 1.16, 95% CI: 0.28–4.86, *P* = 0.83). Participants living with family had 1.65 times higher odds of good understanding (OR: 0.605, 95% CI: 0.12–3.13, *P* = 0.55). Tribhuvan University students had 2.11 times higher odds of good understanding (OR: 0.473, 95% CI: 0.092–2.442, *P* = 0.33) than those from other universities. Most participants from Bagmati province and urban areas had moderate to poor understanding – 95.7% and 96.2%, respectively –, with no statistically significant differences observed across other variables such as income, parents’ profession, or religion (Table 3).

**Table 3:** Factors affecting the understanding of participants about the self-medication of OTC drugs

S.N.	Characteristics	Understanding		Binary logistic regression		
		Moderate to poor (%)	Good (%)	OR	95% CI	*P*
1	Age (Years)					
	Upto 23	28 (87.5)	4 (12.5)	1 (Ref)	0.071–1.282	0.088
	Above 23	93 (95.9)	4 (4.1)	0.301		
2	Gender					
	Male	65 (94.2)	4 (5.8)	1 (Ref)	0.277–4.856	0.830
	Female	56 (93.3)	4 (6.7)	1.16		
3	Living status					
	With family	78 (92.9)	6 (7.1)	1 (Ref)	0.117–3.127	0.545
	Alone	43 (95.6)	2 (4.4)	0.605		
4	University					
	Tribhuvan University	71 (92.2)	6 (7.8)	1 (Ref)	0.092–2.442	0.326
	Others	50 (96.2)	2(3.8)	0.473		
5	Religion					
	Hindu	111 (93.3)	8(6.7)	1 (Ref)	0.889–0.979	0.397
	Others	10 (100)	-	0.933		
6	Monthly household income					
	<50 000	52 (98.1)	1 (1.9)	1 (Ref)	0.629–44.213	0.090
	>50 000	69 (90.8)	7 (9.2)	5.275		
7	One of the parent’s profession					
	Medical	19 (90.5)	2 (9.5)	1 (Ref)	0.105–2.979	0.490
	Non-medical	102 (94.4)	6 (5.6)	0.559		
8	Permanent address					
	Bagmati	45 (95.7))	2 (4.3)	1 (Ref)	0.344–9.178	0.488
	Others	76 (92.7)	6 (7.3)	1.77		
9	Area (Permanent address)					
	Urban	75 (96.2)	3 (3.8)	1 (Ref)	0.620–11.910	0.170
	Rural	46 (90.2)	5 (9.8)	2.71		
10	Understanding					
	Medicine	71 (92.2)	6 (7.8)	1(Ref)	0.09–2.44	0.362
	Others	50 (96.2)	2 (3.8)	0.473		

Participants up to 23 years had 100% moderate to poor practice. Older participants had 1.03 times higher odds of good practice (OR: 1.03, 95% CI: 0.996–1.069, *P* = 0.31). Males demonstrated a slightly better practice (2.9%), with 1.7 times higher odds of good practice (OR: 0.37, *P* = 0.38). Those living with family had better practice (3.6%), though the odds were not significant (OR: 1.91, *P* = 0.52). Students from universities other than Tribhuvan University had 1.5 times higher odds of good practice (OR: 0.568, 95% CI: 0.050–6.423, *P* = 0.643). Hindus had 2.5% good practice with minimal odds difference (OR: 0.97, 95% CI: 0.95-1.003, P-value 0.61). Participants with incomes below 50,000 had better practice (3.8%) (OR 0.34, 95% CI: 0.030-3.849, P-value of 0.362) (Table 4).

**Table 4:** Factors affecting the practice of participants about the self-medication of OTC drugs.

S.N.	Characteristics	Practice		Binary logistic regression		
		Moderate to poor (%)	Good (%)	OR	95% CI	*P*
1	Age (Years)					0.314
	Up to 23	32 (100)	-	1(Ref)	0.996–1.069	
	Above 23	94 (96.9)	3 (3)	1.032		
2	Gender					0.643
	Male	67 (97.1)	2 (2.9)	1 (Ref)	0.050–6.423	
	Female	59 (98.3)	1 (1.7)	0.568		
3	Living status					0.200
	With family	81 (96.4)	3 (3.6)	1 (Ref)	0.925–1.005	
	Alone	45 (100)	-	0.964		
4	University					
	Tribhuvan university	76 (98.7)	1 (1.3)	1 (Ref)	0.268–34.42	0.346
	Others	50(96.2)	2(3.8)	3.04		
5	Religion					
	Hindu	116 (97.5)	3 (2.5)	1 (Ref)	0.947–1.003	0.611
	Others	10 (100)	-	0.975		
6	Monthly household income					
	<50 000	51 (96.2)	2 (3.8)	1 (Ref)	0.030–3.849	0.362
	>50 000	75 (98.7)	1 (1.3)	0.340		
7	One of the parent’s profession					
	Medical	21 (100)	-	1 (Ref)	0.996–1.062	0.440
	Non-medical	105 (97.2)	3 (2.8)	1.029		
8	Permanent address (Province)					
	Bagmati	45 (95.7)	2 (4.3)	1 (Ref)	0.025–3.149	0.271
	Others	81 (98.8)	1 (1.2)	0.278		
9	Area (Permanent address)					
	Urban	76 (97.4)	2 (2.6)	1 (Ref)	0.067–8.606	0.824
	Rural	50 (98.0)	1 (2.0)	0.760		
10	Practice					
	Medicine	76 (98.7)	1 (1.3)	1 (Ref)	0.27–34.42	0.346
	Others	50 (96.2)	2 (3.8)	3.040		

In terms of understanding, 7.8% of medicine students had good understanding compared to 3.8% from other faculties with 2.11 times higher odds of good understanding than other faculties (OR: 0.473, 95% CI: 0.092–2.442, *P* = 0.362). Regarding practice, 1.3 % of participants from medicine and 3.8% from other faculties exhibited good practice. The odds of good practice in other faculties were 3.04 times higher (OR: 3.040, 95% CI: 0.27–34.42, *P* = 0.346). Faculty type did not significantly influence respondents’ understanding or practice of self-medication with OTC medicines.

None of the characteristics examined had a statistically significant impact on participants’ understanding and practice of self-medication (*P* > 0.05 for all variables).

**Figure 1.**
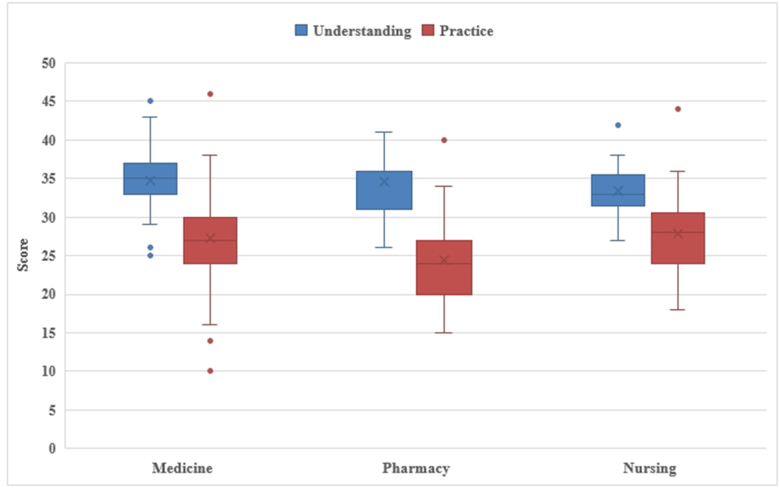
Box plot showing the distribution of understanding and practice scores based on faculties.

The box plot displayed that the understanding scores were generally greater than practice scores across all the faculties (Fig. 1).

There was no significant correlation between understanding and practice scores (Spearman’s rho: 0.114, *P* = 0.197).

**Figure 2.**
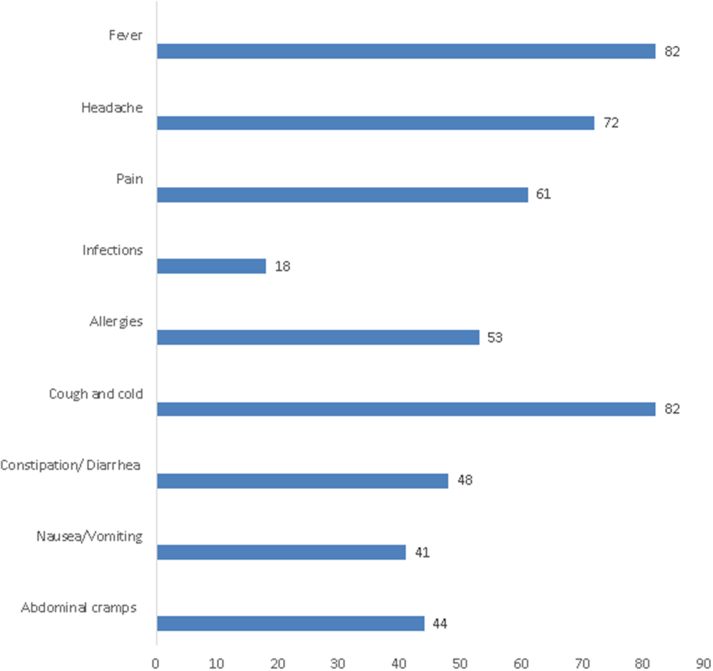
Reasons (Illnesses) for self-medication with OTC.

The key reasons for self-medication with OTC agents were fever and cough/cold, followed by headache and pain (Fig. 2).

**Figure 3.**
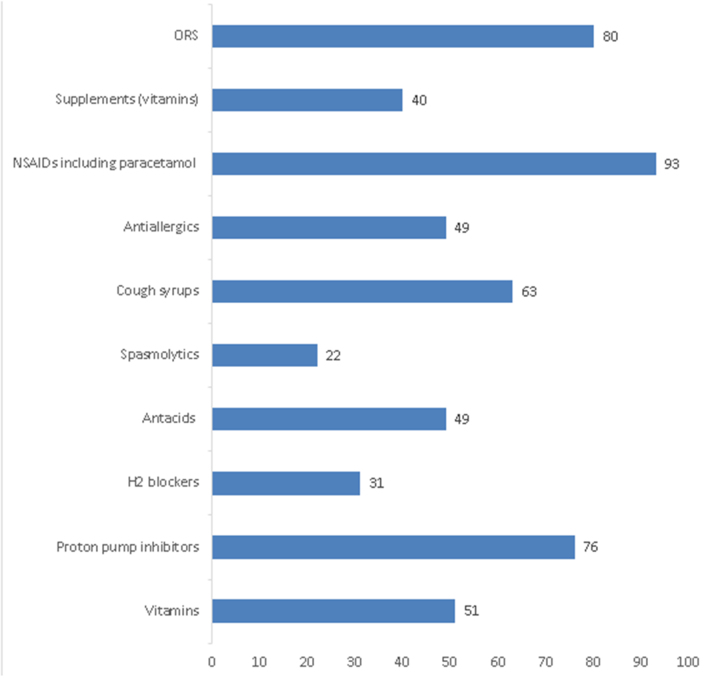
Commonly self-medicated OTC drugs.

The most commonly self-medicated OTC drugs among the respondents were NSAIDs, including paracetamol, followed by oral rehydration solutions (ORS) and proton pump inhibitors (PPIs). H_2_ blockers and spasmolytics were the least frequently used (Fig. 3).

### Discussion

OTC medications play a vital role in the healthcare system, serving as a primary means of addressing common health issues. With rising healthcare costs, the use of OTC drugs and self-medication has significantly increased among both men and women over recent decades^[23]^. In Nepal, the Drugs Act established by the Government of Nepal regulates substances by evaluating their medical applications, the potential for abuse, safety, and risk of dependence. For health science students, familiarity with the Drug Act is essential to ensuring that they are not only knowledgeable about the legal aspects of drug use but also understand the consequences of improper self-medication.

A 2018 study by Hashemzaei *et al* found that self-medication was more prevalent among male students, a trend consistent with our study^[24]^. However, their study reported higher levels of drug knowledge among pharmacy students, which contrasts with our findings showing greater understanding among students from medicine faculty^[24]^. This incongruity might be attributed to variances in curricula and academic years.

A study conducted in 2020 reported a higher prevalence of self-medication amongst students with healthcare professionals in their families, attributing this to increased confidence gained from familial medical knowledge^[15]^. However, our study presents contrasting results, potentially due to the participants living away from their families during their studies. Moreover, a significant proportion of medicine (42.9%), pharmacy (39.1%), and nursing (27.6%) students preferred to recommend OTC drugs to friends or family members, aligning with findings from Akici and Basaran’s study on 205 university students, where 31% recommended medications to relatives^[25]^. This is concerning as health science students, as future healthcare providers, should be advocating against the unadvised sharing of medications and setting a responsible example.

Similarly, a study conducted by Goyal *et al* in 2018 in India found that the use of OTC drugs was widespread among rural populations^[12]^. This is comparable to the results of the present study where good practice was found comparatively low in respondents who were originally from rural areas. However, the understanding level was higher among such respondents. This might be attributed to the urban population’s preference for seeking direct care from hospitals or physicians, while rural populations may resort to self-medication due to the distance from healthcare facilities and associated costs. Additionally, being health science students, our participants’ knowledge of medicine could influence their behavior.

In contrast to a Nigerian study, which identified significant associations between self-medication and factors, such as age, gender, and college, our study found no statistically significant impact of demographic variables on self-medication understanding and practices^[26]^. This suggests that behaviors related to self-medication may be driven by factors other than socio-demographic characteristics. Our findings align with those of a study conducted in Pakistan, where demographic factors were also not found to influence self-medication behavior^[27]^.

Further, a study in Guntur, India found that 78.1% of pharmacy students considered the drugs they used for self-medication to be safe^[19]^. Similarly, our study showed that 52.5% of pharmacy participants perceived OTC drugs as safe for self-care.

In our study, 39% of medicine students disagreed that undergraduate health science students have sufficient ability to diagnose symptoms, while 39.1% of pharmacy students were neutral, and 44.8% of nursing students agreed. This differs from a study in Ethiopia, where most pharmacy students believed health science students had good self-medication abilities^[18]^. Additionally, our study revealed that 29.9% of medicine students agreed that undergraduate health science students should be allowed to self-medicate with OTC drugs, while 39.9% of pharmacy students disagreed, and 37.9% of nursing students remained neutral. By comparison, a separate study reported that 37.3% of respondents agreed with the acceptability of self-medication for health science students^[18]^. These differences may stem from varying sample sizes and study populations.

Though none of the characteristics showed a statistically significant impact on the understanding and practice of participants about self-medication with OTC drugs, higher odds of good understanding and practice were found among males, Hindus, and participants from Bagmati province and urban area. A good understanding was evident among elder participants, those living with family, from Tribhuwan University, having higher family income, and having parents from non-medical backgrounds. Whereas, younger participants, those living alone, from other Universities, having a lesser family income, and having parents of medical backgrounds demonstrated good practice. The faculty type did not significantly influence the understanding or practice of self-medication among the participants. However, medical students had a good level of understanding and the students from pharmacy and nursing faculties demonstrated good practice. The reason behind such a variation might be due to the differences in the nature of the curriculum and the university, family, and the surrounding environments.

Our study also highlighted poor knowledge regarding self-medication, consistent with other research on university students in Portugal^[28]^. This may be linked to outdated curricula that lack information on self-medication. Conversely, studies in Ethiopia have demonstrated good knowledge of self-medication among health science students^[4]^. Similarly, a study in Jordan found that pharmacy students had more potential to practice self-medication than medicine students, mirroring our findings^[15]^. This could be due to the perception among pharmacy students that they possess sufficient knowledge, even though this may lead to unsafe practices.

**Figure 4.**
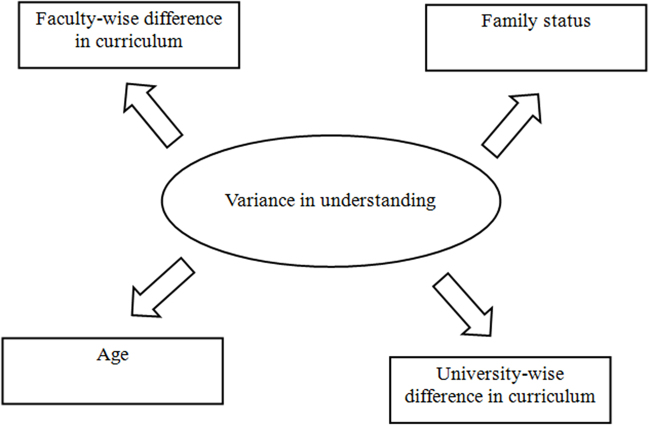
Reasons for variance in understanding.

The variance in understanding among the students of different faculties and universities might be attributed to the differences in curriculum based on faculties and universities, age, and family status including the living status of the participants. The uniformity in the nature of the curricula based on the faculties and the regular updates is required to streamline the understanding of students from different faculties of health sciences (Fig. 4).

A study by Jawahir and Aziz found that 49.9% of respondents kept leftover medications for future use^[29]^. Similarly, in our study, 42.9% of medicine, 52.2% of pharmacy, and 27.6% of nursing students reported self-medicating with leftover OTC drugs. This could be due to the convenience of not needing to purchase new medications, though keeping leftover medications is not recommended. In another study, 71.3% of students reported checking the expiry date of OTC drugs before use, comparable to the 93.4% of students in our study who reported the same, with 39.4% of medicine, 56.5% of pharmacy, and 44.8% of nursing students agreeing to this practice^[19,30]^.

**Figure 5.**
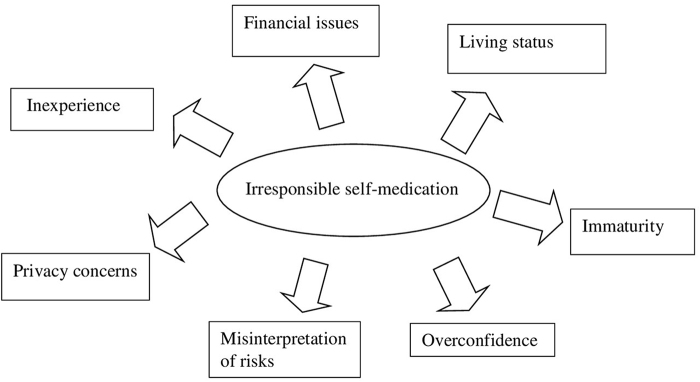
Reasons for irresponsible self-medication practice.

In terms of the most common reasons for self-medication, our respondents frequently cited fever and headache, with NSAIDs being the most commonly used drugs. This is consistent with findings from studies by Kiron, *et al* and Bekele, *et al*^[14,16]^. It is often assumed that health science students, given their exposure to medical knowledge, would practice responsible self-medication. However, our study found that this was not the case, suggesting that their immaturity and inexperience, coupled with overconfidence, may lead to irresponsible practices. Factors such as stress, privacy concerns, financial issues, and a misinterpretation of the risks associated with drug interactions and side effects may also contribute (Fig. 5).

Self-medication has both benefits and risks. While responsible self-medication can be effective for managing minor illnesses, improper use can have serious consequences. Despite the safety of many OTC drugs, misdiagnosis and misuse due to a lack of knowledge about drug interactions and side effects can result in significant harm. Individuals who have prior knowledge of safe and appropriate OTC medications for their condition can make informed decisions and manage such symptoms independently. It is essential to exercise caution and ensure that self-medication practices are based on accurate information. Professional consultation should be sought if symptoms persist or worsen, or if there are signs of a more serious underlying condition.

### Strengths and limitations of the study

This study was the first of its kind conducted in LMICs like Nepal, involving a multicenter approach across various faculties to provide a broader representation of the population. It aimed to identify interdisciplinary gaps and differences in the use of OTC medications. The research addressed a critical public health issue, especially in LMICs, where OTC drugs are widely used for self-care. By focusing on undergraduate health science students, the study offers valuable insights into the understanding and practice patterns of future healthcare providers, potentially guiding future policy and educational reforms.

However, the study is subject to several limitations that warrant consideration. The study encountered sampling bias, as the student sample from medical colleges and universities in Kathmandu may not accurately represent the broader population of Nepal or other LMICs. Time constraints also contributed to these limitations. Response bias may have been present, with participants potentially providing inaccurate or socially desirable answers, especially regarding sensitive questions. Additionally, as a cross-sectional study, it nets data at a single point in time, restricting the ability to evaluate the variation with time.

While the findings may not be fully generalizable worldwide, they offer valuable insights into the broader discussion on OTC drug use and self-medication practices. Future research in similar contexts can validate and expand on our findings, contributing to a deeper understanding of these practices.

We recognize the limitation of grouping participants’ knowledge and practices into “good” and “moderate-to-poor” categories. While this method simplifies data analysis, it may overlook the finer details of individual variations. We suggest using more detailed classification methods or continuous scoring systems in future research to better capture the full range of knowledge and practices. This approach could provide a clearer understanding of gaps and trends, enabling the development of more focused and effective interventions.

### Recommendations

- Health science students should be aware of the consequences of improper use of medications leading to toxicity, increased side effects, and exaggeration of diseases.
- It is crucial to bring attention to the problem of self-medication and to further enhance students’ understanding and practice towards it to empower the new generations against unregulated self-medication.
- The institute’s faculty should inform the students about the benefits and drawbacks of self-medication.
- Education curricula need an upgrade and they must contain information on self-medications.
- More strict policies and strategies must be implemented to regulate self-medication and the sale and distribution of OTC drugs.
- Dissemination of responsible self-medication concepts among health science students through seminars, workshops, and other activities is highly recommended to improve their understanding and practice. This will have a positive influence on the rational use of OTC drugs that bring high safety to medication use.
- More multi-center research on health science students and the general public is required to examine the several elements that affect self-medication.
- Periodically, these studies ought to be carried out so that they will give insight into changing patterns of drug use in society.

## Conclusion

The study revealed that the respondents had a poor understanding of self-medication, which increases the risk of irresponsible and inadequate use of OTC drugs. Self-medication was prevalent among health science students in LMICs like Nepal despite the differences in faculties. Serious issues were identified, such as the use of leftover OTC medicines, neglecting to check expiry dates, and self-medicating due to the proximity of a pharmacy. Fever and cough/cold were the most frequently reported conditions prompting self-medication, while NSAIDs and ORS were the most commonly used medications.

The findings highlight the need for stringent legislation, adherence to the Drug Act, and the effective enactment of programs and guidelines to mitigate irresponsible self-medication practices among health science students. As future healthcare professionals and role models for their peers, health science students have a significant responsibility to set an example in the responsible use of medicines.

## Data Availability

The data is available through the corresponding author on reasonable request.

## Research registration unique identifying number (UIN)

Researchregistry10617.
